# Visuomotor adaptation and savings to constant and varying visual feedback delays in a driving simulator

**DOI:** 10.1167/jov.26.4.7

**Published:** 2026-04-08

**Authors:** Sam Beech, Danaë Stanton Fraser, Iain D. Gilchrist

**Affiliations:** 1 School of Psychological Science, University of Bristol, Bristol, UK; 2 Department of Psychology, University of Bath, Bath, UK; 3 Department of Health & Kinesiology, University of Utah, Salt Lake City, UT, USA

**Keywords:** delay adaptation, savings, delayed visual feedback, visuomotor control, visuomotor adaptation

## Abstract

Perturbations to visual feedback disrupt one's ability to use vision to guide movement, leading to disrupted visuomotor control. The visuomotor adaptation mechanism recovers control by updating the visuomotor mapping to accommodate the visual perturbation during movement. A hallmark of adaptation is savings, where individuals demonstrate faster adaptation upon subsequent exposure to the same perturbation. Although faster adaptation to a previously experienced delay has been observed in response to constant visual feedback delays in two-dimensional tracking tasks, they have not been investigated in ecologically relevant contexts where individuals perform more complex visuomotor control tasks with varying delays. Previously, delay variability has been shown to significantly impair performance within these tasks, but it remains unclear how delay variability will impact adaptation and savings. Therefore, we investigated adaptation to constant and varying delays in a driving simulator over four sessions spaced 7 days apart. Across these sessions, participants exhibited savings, reflected in reduced average absolute spatial error, a shift in the average directional road position toward the middle of the road (instructed position), and flatter learning slopes, indicating a faster approach to asymptote. Crucially, there were no significant differences between the constant and varying delay conditions in any measure. Therefore, participants adapted to the delayed visual feedback with increased efficiency upon subsequent exposure to the same temporal perturbation. Additionally, delay variability did not disrupt adaptation or savings within the driving simulator task.

## Introduction

Effective motor control relies on visually processing the environment to identify the motor commands necessary to achieve a desired outcome (Mehta & Schaal, (2002); Sober & Sabes, 2003; Sober & Sabes, 2005). Perturbations to sensory feedback disrupt this process by impairing the performer's ability to identify the appropriate motor command, resulting in inaccurate movements and degraded motor control that triggers adaptation (Shadmehr, Smith, & Krakauer, 2010). Adaptation to visual disruptions—herein termed visuomotor adaptation—has been studied primarily in response to spatial perturbations, in which the observed direction of movement is displaced from the true direction. The classic paradigm involves using prism goggles that laterally shift visual feedback relative to head orientation (Helmholtz, 1867). When first exposed to this perturbation, participants exhibit pointing errors proportional to the magnitude of the spatial displacement (e.g., a 15° leftward visual shift produces a 15° leftward pointing error), reflecting an inability to compensate for the visual shift. Over time, however, these spatial end point errors are used to update the motor plan and support accurate pointing. When the prisms are removed, participants point in the opposite direction of the initial prism-displacement—this after-effect demonstrates how the visuomotor mapping was updated to accommodate the spatial visual shift (Welch, 2013). More recently, visuomotor adaptation research has expanded to temporal perturbations, in which the visual feedback depicting the movement is delayed. This work is motivated by real-world contexts in which delays between user actions and observed outcomes are common, such as video games (Claypool & Claypool, 2006), virtual reality systems (Caserman, Martinussen, & Göbel, 2019; Waltemate et al., 2016), and teleoperation environments (Sheridan, 1993; Xu et al., 2014). However, existing experimental paradigms fail to capture the complexity of these real-world contexts.

In user-operated technologies, delays occur as the input on the controller must be processed before being rendered on the display. For example, when using slow motion capture, we previously observed a ∼90 ms delay between mouse and cursor movement in a standard laptop setup (Beech, Stanton Fraser, Corston-Petrie, Gower, & Gilchrist, 2024). This example highlights a familiar yet imperceptible delay, but more complex technologies often present larger delays caused by two broad sources: system latency and network latency. System latency refers to the delay within a single device and is determined by the efficiency (Warburton, Mon-Williams, Mushtaq, & Morehead, 2023) and combination of the activated processing components (Georg, Feiler, Hoffmann, & Diermeyer, 2020). When data are passed between devices using a network connection, the additional network latency comprises delays owing to the size of the data packets, the distance the data must travel, network congestion, geographical and weather interference, server storage capacity limits, and data rerouting (Goonatilake & Bachnak, 2012; Nikravesh, Choffnes, Katz-Bassett, Mao, & Welsh, 2014; Noguera Cundar, Fotouhi, Ochitwa, & Obaid, 2023; Stornig, Fakhreddine, Hellwagner, Popovski, & Bettstetter, 2021). Combined, system and network latency create much larger >100 ms delays (Beech et al., 2024). Critically, these factors are not fixed but continuously fluctuate (Georg et al., 2020; Goonatilake & Bachnak, 2012; Jota, Ng, Dietz, & Wigdor, 2013), causing moment-by-moment variability in the delay. Additionally, these technologies often involve complex visuomotor tasks, performed across multiple weeks of exposure (Hing, Sevcik, & Oh, 2009; Ribeiro et al., 2021; Van Rooij, Schoenmakers, Vermulst, Van Den Eijnden, & Van De Mheen, 2011). Therefore, three key components comprise real-world delay exposure: high movement complexity, variability in the delay, and prolonged exposure to the delay. Although adaptation to delayed visual feedback has been investigated in tasks that appropriately simulate one of these components, there has been no investigation using tasks in which all three are simulated.

The existing delay adaptation literature has largely focused on two-dimensional (2D) laboratory-based tasks in which participants control the position of an on-screen cursor along one or both *x* and *y* -axes. Common paradigms include linear target acquisition (Beech, Fraser, & Gilchrist, 2025; Botzer & Karniel, 2013), horizontal target tracking (Cattan, Perrier, Bérard, Gerber, & Rochet-Capellan, 2018; Cunningham, Billock, & Tsou, 2001; Rohde, Van Dam, & Ernst, 2014), and interception tasks (Cámara, de la Malla, López-Moliner, & Brenner, 2018; de la Malla, López-Moliner, & Brenner, 2012; de la Malla, López-Moliner, & Brenner, 2014). This body of research has consistently characterized delay adaptation as a three-phase process. At first exposure, participants assume temporal synchrony between the motor action and the visual feedback, which causes systematic overshooting. The participants terminate their action when the displayed hand appears to reach the target. However, as the visual feedback is delayed, the observed hand continues to move past the target as it catches up with the now static real hand. The spatiotemporal error between the expected and observed hand position is used to refine an estimate for the delay (Rohde & Ernst, 2016). Once a precise estimate is achieved, participants can implicitly predict the current (non-delayed) spatial state of the hand by integrating delayed visual feedback with proprioceptive input and the delay estimate (Botzer & Karniel, 2013; Rohde et al., 2014). When the delay is removed, this predictive update is retained and manifests as undershooting. Participants terminate movement before the observed hand reaches the target, compensating for the delay. However, because the delay is no longer present, the physical hand immediately stops.

To the authors’ best knowledge, investigation of delay adaptation within a complex visuomotor control task that reflects real-world technology is limited to constant delay conditions within driving simulator tasks (Cunningham, Chatziastros, Von der Heyde, & Bülthoff, 2001; Rahimian, Plumert, & Kearney, 2021). In these tasks, participants drive a vehicle through a series of streets while a fixed delay is imposed between their steering inputs and the corresponding update in the virtual vehicle's heading direction. Unlike 2D laboratory-based tasks, the position of the car is not determined by the immediate position of the computer mouse on a 2D plane with a one-to-one cursor mapping, but instead emerges through the continuously evolving speed and steering angle of the vehicle in three-dimensional space. At first exposure, there is a large degree of spatial error between the car and the center of the driving lane as the participants cannot compensate for the delay when controlling the vehicle. With repeated exposure, this continued error feedback is used to update the motor plan to accommodate the delay, leading to decreased spatial error. When the delay is removed, spatial error increases relative to the pre-exposure baseline. Although these purely performance-based metrics do not show exactly how the motor plan was updated, they demonstrate adaptation through recovered performance when exposed to the delay and a pre- to post-exposure decline in performance that signals an updated visuomotor mapping.

As noted, delays in real-world contexts are not constant, but vary continuously in response to dynamic environmental and system factors. We recently demonstrated that participants could adapt to such variable delays. Specifically, participants were exposed to either a constant delay (167 ms) or a temporally varying delay (167 ± 100 ms) during a 2D target acquisition task, in which they used a computer mouse to guide an on-screen cursor toward randomly positioned targets. In both conditions, participants initially overshot the targets, gradually reduced overshooting with increased delay exposure, and then undershot the targets when the delays were removed—patterns consistent with delay adaptation in tracking (Rohde et al., 2014) and reaching tasks (Botzer & Karniel, 2013). There were no significant differences in the adaptation rates or kinematic changes between the constant and varying delay conditions, indicating that delay variability did not disrupt adaptation. However, these findings may not be generalizable to more complex tasks. Within this acquisition task, delay variability had little impact on performance, because increases in variability at various mean delays produced minimal changes in completion time (Beech et al., 2024). In contrast, substantial variability-induced impairments are observed in complex tasks, such as video games (Beznosyk, Quax, Coninx, & Lamotte, 2011) and driving simulators (Davis, Smyth, & McDowell, 2010; Liu, Kwak, Devarakonda, Bekris, & Iftode, 2017; Vozar, Storms, & Tilbury, 2018). In particular, driving shows high sensitivity to delay variability, with participants demonstrating superior control under a constant 358 ms delay than under a varying delay averaging 98 ms (Liu et al., 2017). Therefore, given that delay variability has a greater influence on visuomotor control within these more complex tasks, a similar disruption to adaptation may be observed.

Inter-task differences in sensitivity to delay variability could be attributed to differences in movement duration, dimensionality, and control structure, which combined decrease the reliability of error mapping to a given movement. In 2D screen-based tasks, movements are discrete and approximately linear, such that delay-induced error is expressed primarily along a single spatial dimension (distance error). Under constant delay conditions, the temporal offset between a given movement and feedback produces a consistent spatial displacement between the cursor and target, allowing the delay to be inferred directly from this displacement (Rohde et al., 2014). When the delay varies, the spatial displacement also varies, but as the movement is confined to one dimension the mean delay can be reliably estimated across movements by averaging this spatial displacement (Beech et al., 2025). In contrast, driving involves continuous control over extended periods, with steering inputs indirectly influencing vehicle position. Delay-induced errors are therefore distributed across multiple task-relevant dimensions, including heading direction and lateral position. Under constant delays, the temporal relationship between steering actions and their effects on vehicle motion remains stable, allowing participants to learn when corrective inputs should be applied. Under variable delay conditions, this relationship continuously changes, preventing temporal errors from being reliably assigned to specific actions. Increased difficulty in reliably assigning observed errors to the motor commands that generated them is known to reduce the rate adaptation (Thoroughman & Shadmehr, 2000; Wei & Körding, 2009; Krakauer, Hadjiosif, Xu, Wong, & Haith, 2019).

Another critical aspect of real-world delayed visual feedback is that individuals interact with the same technologies (e.g., Van Rooij et al., 2011) under similar delay (network) conditions (Bauknecht & Enderle, 2020) across multiple sessions. In spatial adaptation paradigms, repeated exposure to the same perturbation typically produces faster re-adaptation, a phenomenon termed savings (Ebbinghaus, 1885; Leow, De Rugy, A., Marinovic, Riek, & Carroll, 2016; Morehead, Qasim, Crossley, & Ivry, 2015). In classic spatial adaptation tasks involving discrete movements, such as reaching or pointing, errors are expressed primarily at the movement end point and learning occurs largely across successive trials (Krakauer et al., 2019; Shadmehr et al., 2010). Consequently, the initial error and asymptotic performance level remain relatively consistent across sessions and the learning curve can be captured with high resolution. These factors allow for direct comparison between sessions, where savings can be quantified using exponential models in which the b parameter captures the early decay in error (Morehead et al., 2015; Smith, Ghazizadeh, & Shadmehr, 2006). Under this framework, faster relearning across sessions produces steeper learning curves as performance approaches the asymptote from the initial error more rapidly.

However, the exponential approach cannot be applied to tasks involving continuous movement with ongoing feedback, meaning that the behavioral signature of savings differs. In these tasks, participants can adapt and adjust their actions during the movement rather than only between trials. As relearning becomes faster across sessions, a larger proportion of the required adaptation occurs during the first trial, reducing the average trial 1 error. Consequently, this initial trial 1 error declines closer to the asymptote, and there is insufficient resolution in the data to observe the early exponential decay phase. Cattan et al. (2018) demonstrated this effect across multiple sessions of delay adaptation within a tracking task. They reported that an exponential fit could not be applied because the model parameters were under-constrained, and instead quantified learning using linear slopes. Because tracking paradigms reliably elicit delay adaptation (Cunningham, Billock, et al., 2001; Miall & Jackson, 2006; Rohde et al., 2014), systematic changes across sessions can reasonably be interpreted as savings. Across sessions, Cattan et al. (2018) observed progressively lower average tracking error and learning slopes that flattened toward the level of the non-delay control condition. In contrast with discrete movement paradigms, where flatter slopes indicate slower learning, flatter slopes across sessions in continuous feedback tasks can arise because faster adaptation occurs during the first trial, bringing the initial error closer to the asymptote and leaving less error to be corrected across subsequent trials. Therefore, in continuous movement tasks, savings measured using linear slopes manifest as a progressively lower average error and flatter learning slopes as the difference in error between the first and final trial declines. To date, evidence of faster adaptation upon re-exposure to a delay is limited to constant delays within target tracking tasks (Cattan et al., 2018; Foulkes & Miall, 2000; Miall & Jackson, 2006). Therefore, not only does it remain unclear how delay variability influences adaptation within a complex visuomotor control task reflective of real-world technology, but it also remains unclear how the rate of adaptation changes with repeated exposure to either constant or varying delays within such a task.

**Figure 1.**
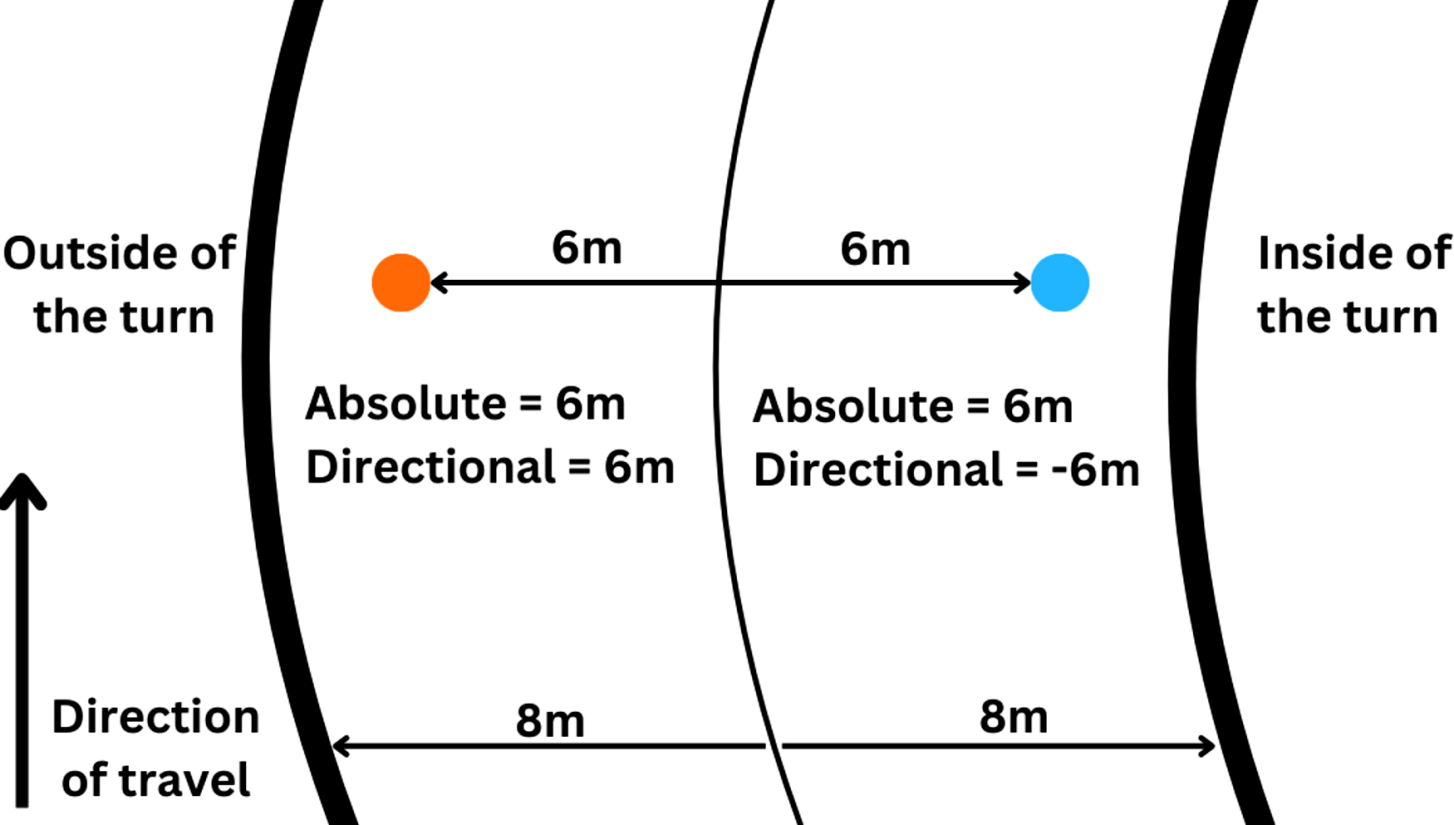
Error metrics. *Note*: The two thick outer lines depict the edges of the road, and the thin center line depicts the center of the road. The orange and blue markers display equidistant vehicle positions from the center of the road on opposite sides. The orange positional marker is placed on the outside of the turn, so absolute and directional error are both 6 m. The blue positional marker is placed on the inside of the turn, so absolute error is 6 m, but directional error is −6 m. The bottom arrows indicate the 16-m width of the road, 8 m for each side.

To address these ecologically relevant questions, we developed a driving simulator to investigate how participants adapted to constant and varying delays across four sessions. The participants were instructed to keep the car as close to the middle of the road as possible, which was represented by two parallel unbroken lines. Although driving in the middle of the road is uncommon in real-world driving, maintaining a car over a line is common in racing video games. Additionally, this instruction provided the participants with an explicit, precise positional reference and error feedback. For each trial, we recorded two performance measures: 1) the average absolute spatial error between the virtual car and the center of the road, and 2) the average directional road position relative to each turn, expressed as the signed distance (in meters) between the car and the road center: negative values indicate a position between the inside of turn and the middle of the road, and positive values indicate a position between the middle of the road and the outside of the turn (Figure 1). The second metric, conceptually similar to horizontal displacement in 2D tracking tasks (see Rohde et al., 2014), served as a directional measure of movement timing: late turns placed the car toward the outside of the turn (positive values), whereas early turns placed it toward the inside of the turn (negative values).

In their driving study, Cunningham, Chatziastros, et al. (2001) defined adaptation as a large spatial error between the car and the assigned driving lane during the first exposure trial, followed by a reduction in this error across subsequent trials. Because Cunningham, Chatziastros, et al. (2001) did not include a directional position measure, we interpret adaptation using conventions from 2D tracking tasks. In such tasks, participants typically overshoot the path on the first trial and gradually learn to maintain a position closer to the intended path (Rohde et al., 2014). Applied to the present driving task, this factor would be reflected as an initial average position toward the outside of the road when the delay is introduced, followed by a shift toward the middle of the road with repeated exposure. Accordingly, we hypothesize that the consistent delay condition will show faster adaptation in session 1, evidenced by a faster decrease in average spatial error and a faster shift in directional road position toward the center of the lane.

Because research investigating delay adaptation across multiple sessions is largely limited to tracking tasks, we use the findings from Cattan et al. (2018) to define savings. Across sessions, their participants showed lower average error and flatter learning slopes. Therefore, in the present task we interpret savings as 1) reduced average absolute spatial error, 2) directional road position closer to the center of the lane, and 3) flatter learning slopes across sessions. Because we expect delay variability to disrupt adaptation, we further predict that the constant delay condition will show significantly faster changes across sessions.

## Methods

Owing to the size of the Unity project file, please email the corresponding author to arrange access to the driving simulator application and all functional scripts. This work was not pre-registered, but the data are available on OSF: https://osf.io/gr2e6/?view_only=30af706d03ec46bea7a5ef46fdebb1eb.

### Participants

Forty-six healthy adult volunteers were recruited, but six did not complete all four sessions so their data were discarded. This resulted in a sample of 40 split evenly between the constant (15 women, 5 men; mean age, 20.3 years; range, 18–27 years) and varying delay conditions (15 women, 5 men; mean age, 22.8 years; range, 18–30 years). The sample size was determined following Latin square randomization of our 10 road layouts, where 20 different road orders were required for a full counterbalance with reversed orders. Participants were required to possess a full driving license from any country and must have normal or corrected-to-normal vision. They were recruited from the student populations of the University of Bristol and the University of Bath, with some non-university participants of a similar age recruited from the Bristol area. They received course credits or a £40 voucher (£10 a session).

### Apparatus and materials

**Figure 2.**
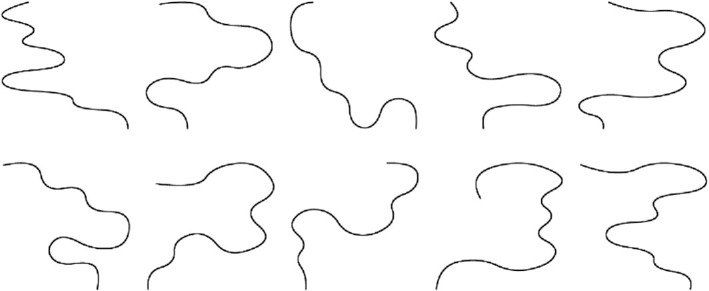
Top–down view of each road. *Note*: The 10 road designs used in the experimental trials. The starting point for each road is the bottom of each line. Each line represents the middle of the road as represented by the tarmac band between the two central white lines in Figure 4.

The task was programmed in the Unity (ver. 2021.3.16f1) game engine using C# scripts. There was a single practice road shaped like a U and 10 experimental road layouts (Figure 2). Like Cunningham, Chatziastros, et al. (2001), the roads were designed using clothoid segments and B-splines to ensure that the change in the optimal steering angle was directly related to the rate of change in the road's curvature. As curvature increased along each turn, the required steering input increased smoothly, reaching a maximum at the point of maximum road curvature before decreasing symmetrically on exit. This ensured smooth transitions in steering inputs and has been proposed as an optimal road design (Lambert, Romano, & Watling, 2019). All 10 experimental roads were 645 to 650 m long and were designed with no change in elevation to ensure that the view of the road remained clear.

**Figure 3.**
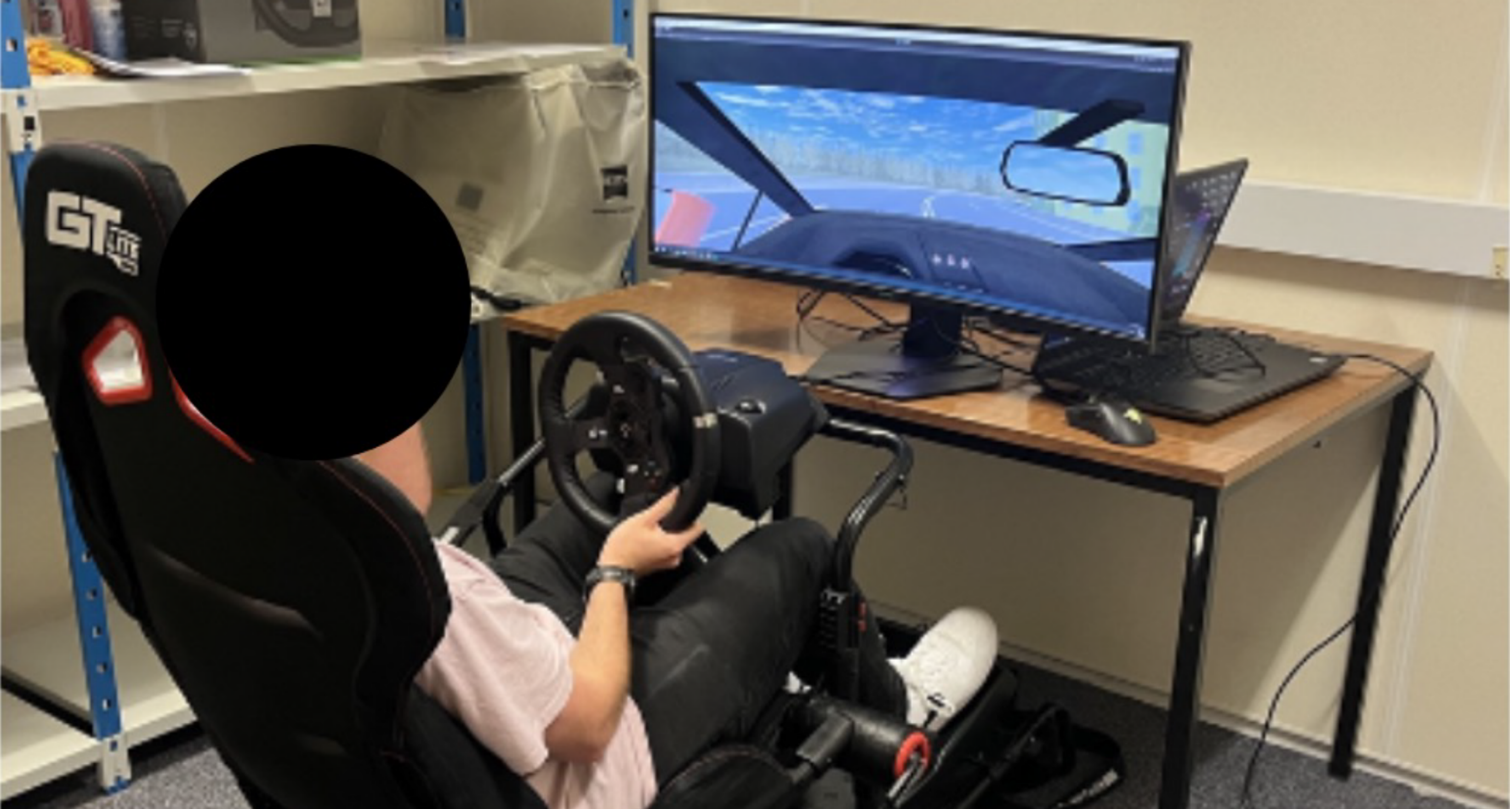
The driving simulator set up. *Note*: The participants completed the experiment with the room light switched off, with the monitor as the single light source.

**Figure 4.**
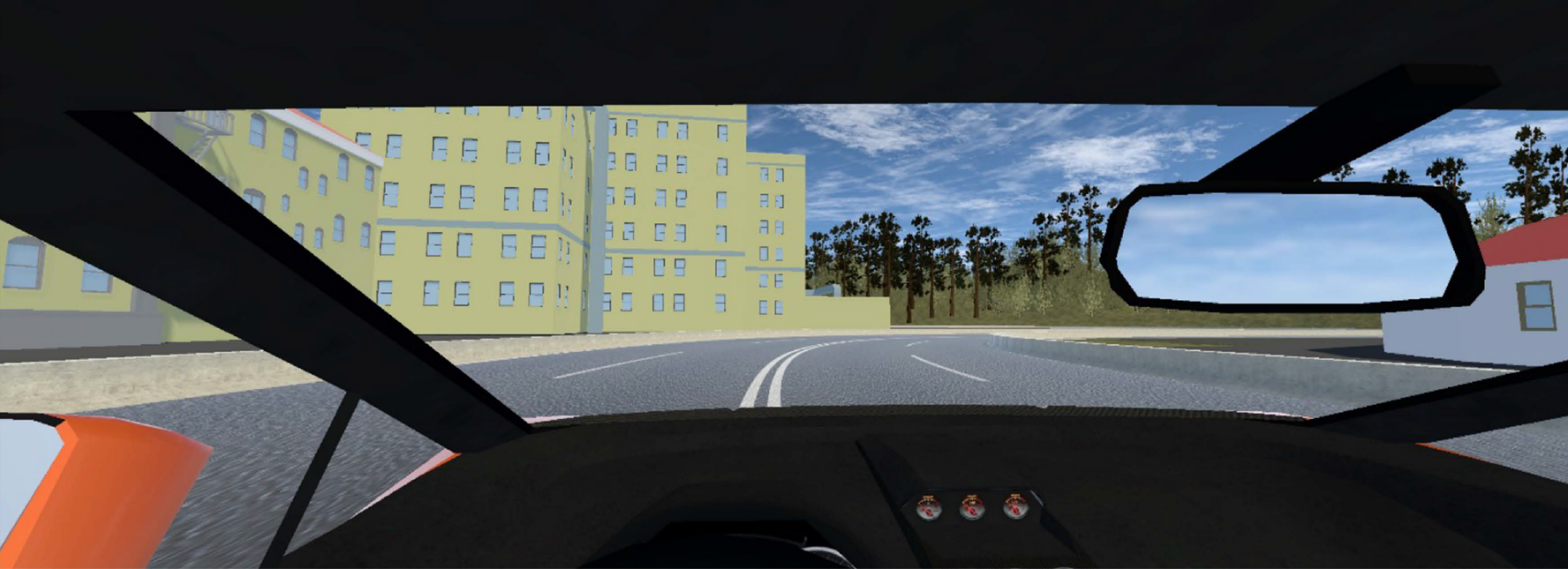
First-person view in the virtual car. *Note*: The view from the virtual car during the first turn of the fourth road. All environmental features outside of the outer road walls are decorative and did not interfere with the view of the upcoming road. The participants were instructed to maintain the car in the center of the road, marked by the solid parallel lines.

The task was presented on a Dell (Dell Technologies, Round Rock, TX) S3422DWG 34-inch (144 Hz, WQHD 3,440 × 1,440 resolution, 80.7 cm × 36.63 cm) curved widescreen monitor. The participants were seated in an adjustable Next Level Racing GTLite foldable simulator (Figure 3). Because participants adjusted the seat to their preferred driving position, there was variability in viewing distance, but a typical viewing distance was approximately 80 cm. Based on the active display area of the 34-inch, 21:9 panel, the horizontal and vertical viewing angles subtended by the display at this distance were 53.2° and 23.6°, respectively, at this distance. The participants observed a first-person view from the virtual driving seat (Figure 4) and controlled the car using a Logitech G920 force feedback steering wheel that provided self-aligning torque. Within the Logitech G Hub software, the steering wheel was set to a sensitivity of 50, a 900° operating angle, and a center spring strength of 20. The rotation of the virtual steering wheel was matched to the Logitech steering wheel. Steering input was sampled as a normalized horizontal axis value and mapped linearly to the vehicle's front wheel angle. The normalized input (mapped as −1 to 1 of the 900° operating angle) was multiplied by a fixed maximum steering angle of ±30° and applied to the front left and right wheels. Vehicle turning behavior was governed by Unity's built-in rigid-body physics using WheelCollider components, whereby lateral tire forces and yaw motion emerge from internally computed slip-based tire dynamics with the ground.

Although limbs are typically hidden from view in visuomotor adaptation tasks, we did not block the view of the hands because this would have partially obstructed the screen. The participants could therefore see the temporal discrepancy between their hand movement and the movement of the virtual car. This factor could impair adaptation because the observed temporal alignment between the motor action and the corresponding visual feedback is not entirely decoupled (see Rohde et al., 2014). However, Cunningham, Chatziastros, et al. (2001) used the same setup and observed adaptation in their driving task. Furthermore, when driving, road users generally have poor perception of their hand positions, despite them remaining within the visual field (Thomas & Walton, 2007). Consequently, the maintained direction of attention toward the virtual road has been shown to be sufficient for delay adaptation.

### Baseline delay

The baseline motion-to-photon delay was not measured in this setup. However, the same laptop was used in previous experiments, where the mean delay was 90 ms with a range of ±5 ms (Beech et al., 2024; Beech et al., 2025). The previous task was programmed in MATLAB and presented on a 60-Hz monitor, whereas the present task was developed in the gaming-specific Unity engine and displayed on a 144-Hz monitor. As a result, the baseline delay in the current setup was likely lower than observed in the previous task and remained similar to the <100 ms delays found in modern human–computer interaction systems (Hadjiosif, Abraham, Ranjan, & Smith, 2024).

### Delay manipulation

The effect of the steering wheel input on the virtual car's heading direction was controlled using C# scripts that interacted with the Unity physics engine. The steering inputs were recorded at 50 Hz and changed the angle of the front wheels. To impose a constant delay, the inputs were buffered for 230 ms before being passed to the steering function. This latency was selected because it is the value where Cunningham, Chatziastros, et al. (2001) observed the greatest adaptation in their driving task. The varying delay condition had an average delay of 230 ms, a range of ±100 ms, and a frequency of 2.5 Hz. Therefore, a new delay value from within this range (integers from 130 to 330 ms) was selected at random every 400 ms, providing a uniform distribution. These values were taken from our previous experiment (Beech et al., 2025) so that differences in adaptation could be reliably attributed to differences in the task.

### Task

The participants were instructed to keep the virtual car (1.8 m width × 4.2 m length) as close to the middle of the road as possible. The road was 16 m wide with walls on either side. If the participants crashed into a wall, the virtual car returned to the start of the road. In the presence of delayed visual feedback, participants often use an explicit strategy where they reduce the vehicle speed to minimize the effect of the delay (Sheridan, 1993). To prevent this, we used the same approach as Cunningham, Chatziastros, et al. (2001), where the speed of the vehicle was automatically controlled using a similar system to cruise control. The virtual car accelerated to 30 mph on the straight before the first turn, the standard speed limit for suburban areas in the UK. The virtual car then maintained this speed, but turning caused slight deceleration owing to friction-based interaction between the tires and ground.

### Design

We used the standard three-phase design for investigating delay adaptation (e.g., Cunningham, Billock, et al., 2001; Cunningham, Chatziastros, et al., 2001). First, the participants completed 10 pre-exposure phase trials with non-delayed visual feedback to set a baseline performance level. In the exposure phase, they completed 20 trials with either a constant or varying delay. In the post-exposure phase, they completed 10 trials with non-delayed visual feedback to test for an after-effect and to washout the learned update. Two measures were used to assess driving performance. The first was the average absolute spatial error between the center of the car and the middle of the road throughout each trial. The second measure was the average directional position of the car on the road relative to each turn throughout each trial, The position of the car relative to the center of the road was recorded at 50 Hz. Although crashes reset the car, they did not reset the recording of each measurement.

### Procedure

The chair and steering wheel were adjusted to the participant's preferred position. They were then informed that they would be driving through a series of roads and that they must stay as close to the middle as possible. They were also informed that there were regular breaks (after roads 6, 16, 26, 36, 46). First, the participants completed an introductory U-shaped road at half-speed (15 mph) to familiarize themselves with how their steering wheel inputs controlled the turning behavior of the virtual car, then moved on to the practice phase where they completed each of the 10 experimental road layouts once at the full 30 mph speed in their counterbalanced order. These trials allowed the participants to experience each road once before data collection began.

In the experimental phase, participants completed 10 trials in the pre-exposure phase with non-delayed visual feedback. The participants were then verbally informed that there would now be a delay between their input on the steering wheel and its effect on the virtual car before completing the 20 exposure phase trials in their assigned delay condition. Upon progression to the post-exposure phase, the participants were told that the delay had been removed before completing the 10 non-delay post-exposure trials. After this, the testing session (∼60 minutes) ended. Across the 50 roads (not including the U-shaped introductory road), the participants completed their assigned counterbalanced road order five times in a row (once in practice, once in the pre-exposure phase, twice in the exposure phase, and once in the post-exposure phase). The participants repeated this task across four sessions, each spaced 7 days apart. They remained in their assigned delay condition and counterbalanced road order in all four sessions.

## Results

The data were analyzed using JASP (JASP Team, 2023). Initially, we planned to fit exponentials to the data to compare the ‘a’ values (initial error) and the ‘b’ values (rate of change/adaptation) for each participant's change in performance throughout the exposure phase. However, these fits were poor for both delay conditions (see the Discussion for further explanation). Cattan et al. (2018) faced similar challenges and instead used linear mixed-effects models (LMMs). Therefore, we also used LMMs to measure changes in performance throughout the exposure phase, with ‘delay condition’ as a categorical fixed effect, ‘trial number’ and ‘session number’ as scalar fixed effects factors, and ‘participant’ specified as a random effects with random intercepts and slopes for the repeated measures, ‘road’ and ‘session,’ and their interactions.

Although the non-delay post-exposure phase in conjunction with the 7-day period between sessions ensured that the participants began each session with the same baseline visuomotor mapping, general improvements in driving simulator ability were expected as participants became more familiar with the mapping between the controller and virtual car. Therefore, to accommodate these improvements when analyzing the exposure phase data, for each session, we calculated each individual participant's average absolute spatial error across the 10 pre-exposure phase trials and then subtracted this value from each of their trials within the exposure phase. This step normalized the exposure phase data so that any differences between sessions could be reliably attributed to changes in adaptation. However, because the average road position was directional (with positive and negative values reflecting differences in direction), this approach could not be applied for this measure.

To investigate changes in the amplitude of the after-effects across the four sessions, we compared the change in the average performance from the pre-exposure phase to the first post-exposure trial using a 2 (delay condition) × 2 (phase) × 4 (session) mixed measures analysis of variance. The use of the first post-exposure trial accounts for adaptation decay that occurs throughout the 10 post-exposure trials.

Finally, although we recorded each participant's number of crashes per road, it was so infrequent throughout the pre-exposure phase (mean, 0.001; range, 0–1), the exposure phase (mean, 0.05; range, 0–3), and the post-exposure phase (mean, 0.004; range, 0–1) that a formal analysis was not completed. Please note that crashing was most frequent on the first trial in the experimental phase (mean, 0.24; range, 0–3) and all other trials averaged 0.1 or fewer crashes per trial.

### Absolute spatial error

**Figure 5.**
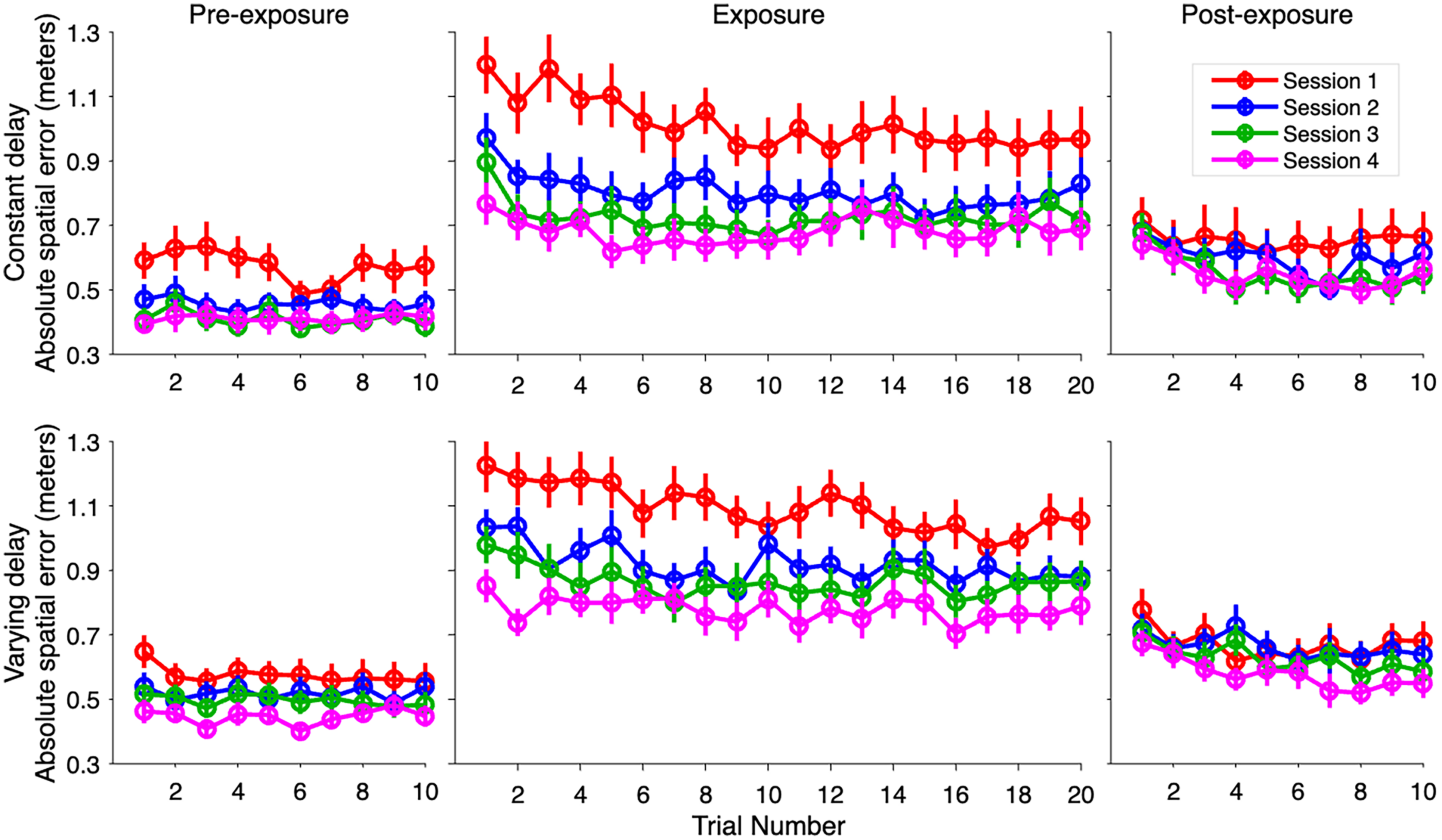
Average absolute spatial error across the four sessions. *Note*: Each datapoint represents the mean spatial error for the constant delay (top) and the varying delay (bottom) for each trial. The error bars show the standard error of the mean. This is the raw observed data and not the normalized exposure phase data.

The average spatial error throughout the four sessions is shown in Figure 5. The change in the absolute spatial error throughout the exposure phase across the four sessions was investigated using an LMM. The difference estimate between the two delay conditions was non-significant, β = −0.03, *t*(37.96) = −0.84, *p* = 0.408, showing no significant difference in the average absolute exposure phase spatial error between the two delay conditions. The slope estimate for the trial number was significant, β = −0.01, *t*(40.32) = −6.50, *p* < 0.001, showing a significant decrease in the absolute spatial error throughout each exposure phase. The slope estimate for the session number was also significant, β = −0.09, *t*(39.81) = −9.22, *p* < 0.001, reflecting an overall decrease in the average absolute spatial error with each session. The two-way interaction between the delay condition and the trial number was non-significant, β = −0.001, *t*(40.32) = −0.39, *p* = 0.696, indicating no significant difference in the average slope between the two delay conditions. The two-way interaction between the delay condition and session number was also non-significant, β = −0.003, *t*(39.81) = −0.27, *p* = 0.792, demonstrating no significant differences in the session-to-session decrease in error between the two delay conditions. The two-way interaction between the session and trial number was significant, β = 0.003, *t*(54.66) = 5.10, *p* < 0.001, showing that the exposure phase slope flattened with each session (Figure 5; Table 1). Finally, the three-way interaction between the delay condition, trial number, and session number was non-significant, β = −0.0004, *t*(54.66) = 0.83, *p* = 0.413.

**Table 1.** The estimated slopes and CIs for the change in absolute spatial error throughout the exposure phase in each session.

Session	Slope	Lower 95% CI	Upper 95% CI
1	−0.010	−0.013	−0.007
2	−0.006	−0.008	−0.003
3	−0.003	−0.006	−0.0001
4	−0.001	−0.003	−0.001

CI, confidence interval.

The change in after-effects across the four sessions was investigated using a 2 (delay condition) × 2 (phase) × 4 (session) mixed measures analysis of variance that compared the change in absolute spatial error from the pre-exposure phase to the first post-exposure trial. The session factor was found to violate Mauchly's test of sphericity, χ^2^(5) = 22.44, *p* < 0.001, so a Greenhouse–Geisser correction was applied to this factor. The main effect of the delay condition was non-significant, *F*(1, 38) = 0.64, *p* = 0.428, $\eta_{p}^{2}$ = 0.02, showing no significant differences between the two delay conditions. A significant main effect of the phase was observed, *F*(1, 38) = 93.03, *p* < 0.001, $\eta_{p}^{2}$ = 0.71, showing a significant increase (0.21 m) in the average absolute spatial error from the pre-exposure phase and first post-exposure trial. A significant main effect of the session was also observed, *F*(2.15, 81.67) = 10.32, *p* < 0.001, $\eta_{p}^{2}$ = 0.21, showing a decrease in the average absolute spatial error with each session. The two-way interactions between the condition and session, *F*(2.15, 81.67) = 0.19, *p* = 0.846, $\eta_{p}^{2}$ = 0.01, the condition and phase, *F*(1, 38) = 0.03, *p* = 0.865, $\eta_{p}^{2}$ = 0.001, and the session and phase, *F*(2.51, 95.27) = 1.74, *p* = 0.173, $\eta_{p}^{2}$ = 0.04, were all non-significant. Finally, the three-way interaction was also non-significant, *F*(2.51, 95.27) = 1.23, *p* = 0.303, $\eta_{p}^{2}$ = 0.03. The results show a significant increase in average absolute spatial error from the pre-exposure phase to the first post-exposure trial, but the size of the after-effects did not differ across each session or between each delay condition.

### Directional road position

**Figure 6.**
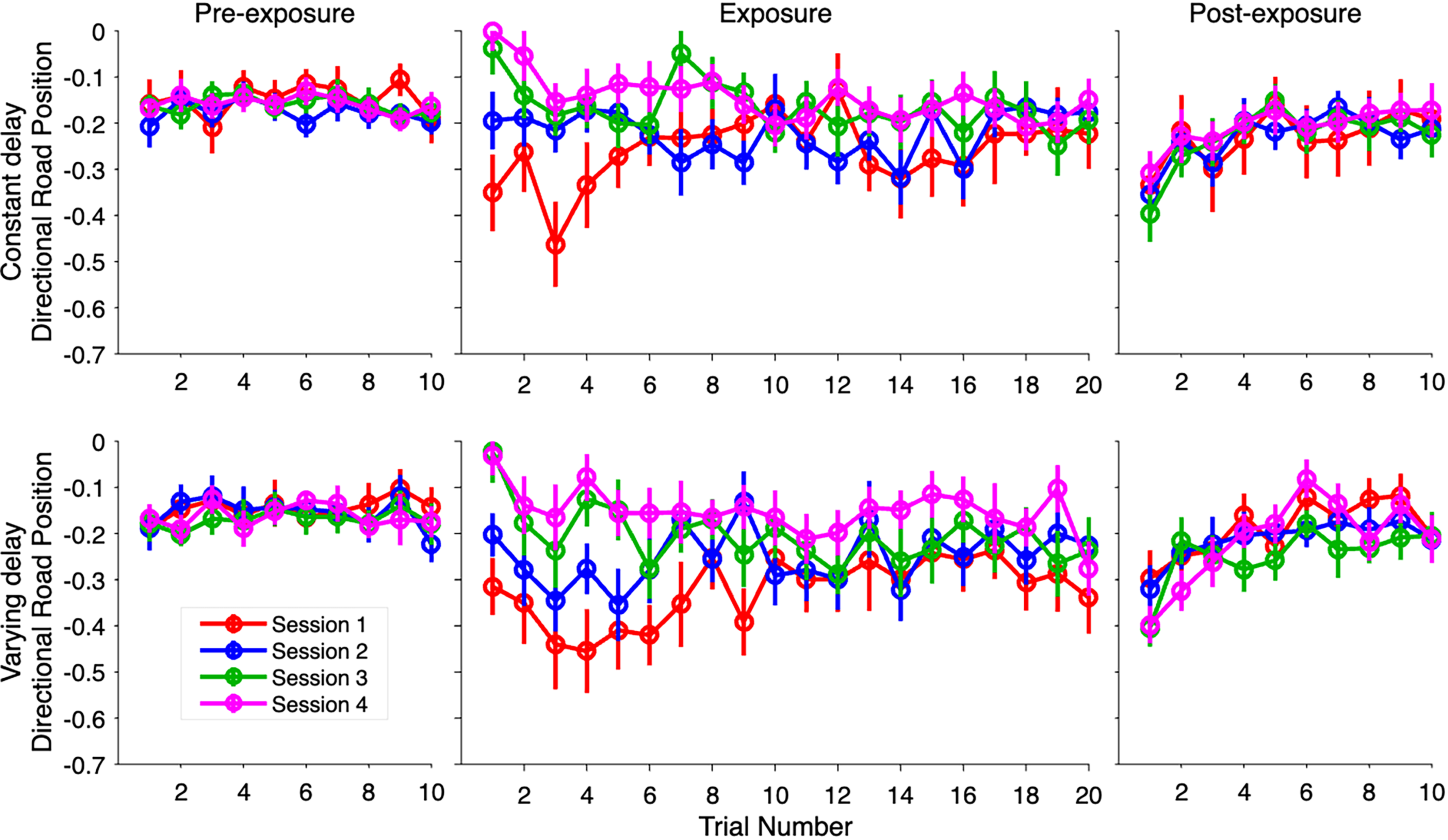
Average directional road position across the four sessions. *Note*: Each data point represents the average directional road position for each delay condition. A positive value represents an average position between the outside of the turn and the middle of the road, and a negative value represents an average position between the middle of the road and the inside of the turn. The error bars represent the standard error.

The average directional road positions throughout the four sessions are shown in Figure 6. The change in the average directional road position throughout the exposure phase across the four session was investigated using an LMM. The difference estimate for the type of delay condition was non-significant, β = 0.05, *t*(25.47) = 1.01, *p* = 0.324, indicating no difference between the two delay conditions. The slope estimate for the trial number was significant, β = 0.01, *t*(44.02) = 4.24, *p* < 0.001, indicating a change in the average directional road position throughout the exposure phase. The slope estimate for the session number was also significant, β = 0.09, *t*(29.59) = 7.08, *p* < 0.001, showing a change in the average directional road position from the inside of the turn toward the middle of the road with each session (session 1 = −0.291 m; session 2 = −0.235 m; session 3 = −0.185 m; session 4 = −0.148 m). The two-way interactions between delay condition and trial number, β = −0.001, *t*(44.02) = −0.54, *p* = 0.594, and condition and session, β = 0.01, *t*(29.59) = −0.79, *p* = 0.433, were non-significant. However, the interaction between the trial number and session number was significant, β = −0.004, *t*(89.96) = −5.48, *p* < 0.001, which provides context to the main effect of trial number and indicates that change in the average road position throughout the exposure phase differed between sessions: the average road position shifted from the inside of the turn toward middle of the road in sessions 1 and 2, but shifted from the middle of the road toward the inside of the turn in Sessions 3 and 4 (Figure 6; Table 2). Finally, the three-way interaction was non-significant, β = 0.0001, t(89.96) = 0.22, *p* = 0.823.

**Table 2.** The estimated slopes and CIs for the change in road position throughout the exposure phase in each session.

Session	Slope	Lower 95% CI	Upper 95% CI
1	−0.006	0.002	0.010
2	0.002	−0.02	0.005
3	−0.005	−0.008	−0.002
4	−0.005	−0.008	−0.001

CI, confidence interval.

The change in the after-effects across the four sessions was investigated using a 2 (delay condition) × 2 (phase) × 4 (session) mixed measures analysis of variance that compared the change in the average directional road position from the pre-exposure phase to the first post-exposure trial. The session factor was found to violate Mauchly's test of sphericity, χ^2^(5) = 20.95, *p* < 0.001. Therefore, a Greenhouse–Geisser correction was applied. The main effect of the delay condition was non-significant, *F*(1, 38) = 0.003, *p* = 0.960, $\eta_{p}^{2}$ = 0.0001, showing no significant difference between the two delay conditions. A significant main effect of the phase was observed, *F*(1, 38) = 53.25, *p* < 0.001, $\eta_{p}^{2}$ = 0.58, showing a significant change in the average directional road position from the middle of the road in the pre-exposure phase (−0.16 m) toward the inside of the turn in the post-exposure phase (−0.35 m). The main effect of the session number was non-significant, *F*(2.39, 90.99) = 2.35, *p* = 0.091, $\eta_{p}^{2}$ = 0.06, showing no significant differences between the sessions. The two-way interactions between session and condition, *F*(2.39, 90.99) = 1.48, *p* = 0.231, $\eta_{p}^{2}$ = 0.04, phase and condition, *F*(1, 38) = 0.03, *p* = 0.871, $\eta_{p}^{2}$ = 0.0007, and session and phase, *F*(2.68, 101.83) = 1.89, *p* = 0.142, $\eta_{p}^{2}$ = 0.05, were all non-significant. The three-way interaction was also non-significant, *F*(2.68, 101.83) = 1.37, *p* = 0.256, $\eta_{p}^{2}$ = 0.04. The results demonstrate a significant change in road position toward the inside of the turn from the pre-exposure phase to the first post-exposure trial. This effect was consistent between the two delay conditions across all four sessions.

## Discussion

This experiment aimed to determine whether delay variability disrupts adaptation and savings in a driving simulator task. Throughout the first session, participants adapted and recovered visuomotor control, evident through a decrease in the average absolute spatial error and a shift in the average directional road position from the inside of the turn toward the center—although this was in the opposite direction to what was expected. Across sessions, the savings effect was observed as a decline in the average absolute spatial error, a shift in the average directional road position toward the center, and flatter learning slopes in both measures. However, contrary to our hypothesis that delay variability would disrupt adaptation and savings, there were no significant differences between the constant and varying delay condition in any recorded measure.

Previously, varying delays have been shown to substantially impair driving performance (Davis et al., 2010; Liu et al., 2017; Vozar et al., 2018), but variability did not significantly disrupt performance, adaptation, or savings in our task. Davis et al. (2010) used a 700 ms mean and ±300 ms range, Vozar et al., 2018 used a 250 ms mean and 125 ms standard deviation, and Liu et al. (2017) used a 95 ms mean and a 55 ms standard deviation. Therefore, the absence of variability-induced impairment cannot be attributed to differences in the perturbation magnitudes, as we applied a mean delay of 230 ms with a ±100 ms range. We initially proposed that the detrimental effects of delay variability reported in earlier studies could be attributed to the complexity of the movements required in driving tasks. However, the absence of variability-induced impairments replicates our 2D target acquisition studies (Beech et al., 2024; Beech et al., 2025), indicating that task complexity alone does not determine sensitivity to delay variability.

The average directional road position data provide insight into why delay variability did not disrupt performance. Despite explicit instruction to maintain the car in the center of the road (0 m), the pre-exposure average position was consistently biased toward the inside of the turn (−0.16 m). This result likely reflects path optimization in driving, where drivers position the vehicle in a subjectively optimal trajectory rather than strictly maintaining the lane center (Lappi, 2014). Cutting the corner increases the effective turning radius, reduces steering input, and smooths the vehicle trajectory by minimizing jerk and speed fluctuations (Itkonen et al., 2017). Although we initially hypothesized that delayed steering would shift the vehicle position toward the outside of the turn owing to lagged control, the opposite pattern was observed. One possible explanation is that maintaining an inside trajectory reduced the amplitude and frequency of corrective steering adjustments, thereby limiting the propagation of control errors under delayed feedback. Under sensorimotor uncertainty, systematic performance errors may be tolerated if they reduce movement costs or improve control stability (Nagengast, Braun, & Wolpert, 2010). This interpretation is also consistent with risk-based models of driving, which show that drivers often position the vehicle closer to the inside of a curve during natural driving (Kolekar, De Winter, & Abbink, 2020). Thus, the observed inside bias likely reflects a strategic path optimization that remained beneficial even under delayed feedback.

In addition to this overall bias, the average road position on the first exposure trial differed across sessions. These differing starting values may reflect strategic adjustments rather than adaptation itself. When encountering delayed steering feedback, participants may initially adopt a cautious control strategy, cutting the corner to reduce the necessary steering input and the likelihood of overshooting the turn. As participants experience the delay within a session, they rapidly recalibrate their steering control, reducing the need for this conservative positioning. This strategy would produce the observed convergence toward baseline behavior during exposure. Under this interpretation, the differing starting values across sessions reflect initial control strategies, whereas the within-session convergence reflects sensorimotor adaptation to the delay. This account is also consistent with the clear after-effects observed in the first post-exposure trial of each session, indicating that an adapted control policy had been acquired during the exposure phase.

Although this behavior may explain why delay variability did not significantly disrupt visuomotor control, it does not explain why the rate of adaptation was unimpaired. It was previously suggested that delay variability should impair adaptation by introducing uncertainty into the error signal available for feedback-based learning (Rohde & Ernst, 2016), where the error for a given movement changes as the moment-to-moment delay magnitude changes. However, we previously proposed that the statistical properties of this uncertainty may determine how delay variability influences adaptation (Beech et al., 2025). Specifically, stationary distributions—where the mean and variance remain constant over time—supports optimal adaptation (e.g., Beech et al., 2025), whereas non-stationary distributions—where these properties fluctuate—impairs learning (e.g., Knelange & López-Moliner, 2019). When adapting, cumulative error signals must be processed to identify the optimal estimate for minimizing movement error (Korenberg & Ghahramani, 2002; Körding & Wolpert, 2004). In stationary distributions, the temporally consistent mean and variance allow the optimal error signal (the mean delay) to be extracted with minimal exposure. The mean delay in the varying delay condition matched the constant delay value, and the common adaptation rate and performance suggests convergence toward a common temporal estimate. This conclusion is supported by Cunningham, Chatziastros, et al. (2001), who showed that different constant delay magnitudes yield different learning rates. In contrast, consistent with signal processing models Widrow, McCool, Larimore, and Johnson (1976), temporal instability of the mean and variance in non-stationary distributions requires prolonged exposure to identify the optimal error-minimizing estimate. The present results provide further support for this hypothesis: our use of a uniform (stationary) distribution did not impair adaptation or performance, whereas significant driving impairments were observed with non-stationary distributions such as tailed distributions (Vozar et al., 2018), Pareto distributions (Liu et al., 2017), and sum-of-sines sequences (Davis et al., 2010). Future work must compare adaptation to varying delays across different distributions within the same task to confirm this hypothesis.

Despite observing a savings effect within the adaptation phase, the after-effects remained consistent across sessions. Although adaptation became increasingly efficient over time, the rate of de-adaptation did not change. Each trial involved ∼50 seconds of continuous driving, and performance was averaged across this period. If participants had become more efficient at de-adapting, we would expect a progressive reduction in average spatial error during the first post-exposure trial across sessions. However, this was not observed in either performance measure. This dissociation aligns with the two-stage model of adaptation (Smith et al., 2006; Zarahn, Weston, Liang, Mazzoni, & Krakauer, 2008), which proposes that motor learning reflects the interaction of a fast process, characterized by rapid learning and forgetting, and a slow process, characterized by gradual learning and longer retention. In particular, the current findings support the hypothesis that savings reflects increasingly efficient action selection mediated by the explicit, fast process (Morehead et al., 2015), whereas after-effects predominantly reflect implicit recalibration governed by the slow process, which remains stable across sessions (Morehead, Taylor, Parvin, & Ivry, 2017).

Although the present task was not intended to simulate real-world driving, there are various changes that are necessary in future work for valid generalization. First, participants did not control vehicle speed. This strategy was implemented to prevent participants from adopting the strategy of driving slowly to mitigate the effects of delay, which can reduce or prevent adaptation (Sheridan, 1993). In natural driving contexts, drivers would likely initially reduce speed in response to delay but subsequently increase speed with experience to meet environmental demands, such as traffic. The timescale over which this occurs remains unknown, and contexts that require speed were not present in the current task. Future work should therefore adopt more naturalistic approaches that ensure maintained speed while preserving task demands. Second, instructing participants to drive in the middle of the road does not reflect typical driving. Simulating such conditions is important for generalizing findings to emerging technologies, such as self-driving vehicles with remote operator override. Third, control of a virtual vehicle lacks the physical consequences associated with real or teleoperated vehicle control. Currently, the influence of stress-related physiological responses, such as increased heart rate and cortisol levels, on visuomotor control under delay conditions remains an important and uninvestigated direction for future research. Fourth, we did not continuously record the virtual cars’ raw positional data for further analysis. This information would have allowed a deeper investigation into the influence of the delays on the cars travel path and is something we will add into our future research. Finally, the task probed only one component of driving—the ability to maintain vehicle position within a desired space—whereas real-world driving involves a range of additional demands, including turning, parking, interaction with other road users, varying road types, and distraction. The task was designed to isolate visuomotor control demands to examine adaptation to constant and varying delays within a more complex control context. The present findings demonstrate adaptation under both delay conditions in a driving simulator and provide a baseline for future work examining delay adaptation across more diverse driving contexts.

One limitation of this study was our inability to use exponential fitting to quantify and compare adaptation rates between sessions. Visuomotor adaptation is reliably modeled using an exponential function, with the learning rate indexed by the ‘b’ parameter, which reflects the rapid early phase of learning (e.g., Hosseini, Nguyen, & Joiner, 2017; Morehead et al., 2015). This approach requires sufficiently high temporal resolution to capture the exponential error decay trend, as well as consistency in both the starting error ‘a’ and asymptotic performance level ‘c,’ such that learning occurs within a standardized range for meaningful comparison of ‘b’ values across sessions (see Murdock & Cook, 1960). These requirements are typically met in traditional visuomotor tasks involving discrete, single-movement trials. In such tasks, an unexpected perturbation produces a consistent starting error ‘a,’ and because the movements are well learned, participants can fully adapt and return to similar asymptotic performance levels ‘c’ across sessions. Moreover, because each trial consists of a single movement, learning can be tracked with high temporal resolution, allowing the fast early decay phase to be captured by the exponential model. In contrast, these conditions are not met in more complex visuomotor tasks involving continuous movement. In the present task, each trial comprised ∼50 second of driving and was represented by a single averaged measure, reducing temporal resolution and preventing capture of the early exponential decay trend. Additionally, as the rate of adaptation improved, this exponential decay was lost in the trial 1 average and manifested as a lower trial 1 average error. Similar limitations have also been reported in continuous tracking tasks (Cattan et al., 2018). Although exponential fitting was unsuitable in the present study, changes in learning rate could still be statistically explored using LMMs, consistent with previous work in delay adaptation contexts.

## Conclusions

Our results demonstrate that participants adapted to constant and varying delays with increased efficiency in a complex driving simulator task. We observed no significant differences between the two delay conditions in the average performance level, adaptation rate, or savings effect. The results suggests that factors other than task complexity determine the impact of delay variability on visuomotor control and adaptation.

## References

[bib1] Bauknecht, U., & Enderle, T. (2020). An investigation on core network latency. *2020 30th International Telecommunication Networks and Applications Conference (ITNAC)*. Virtual Conference, November 25–27, 2020 (pp. 1–6). IEEE, 10.1109/ITNAC50341.2020.9315007.

[bib2] Beech, S., Stanton Fraser, D., Corston-Petrie, A., Gower, A. P., & Gilchrist, I. D. (2024). How changes in the mean latency, jitter amplitude, and jitter frequency impact target acquisition performance. *ACM Transactions on Applied Perception**,* 22(2), 1–18, 10.1145/3701984.

[bib3] Beech, S., Fraser, D. S., & Gilchrist, I. D. (2025). Visuomotor adaptation to constant and varying delays in a target acquisition task. *Journal of Vision**,* 25(6), 8, 10.1167/jov.25.6.8. PMC12118507 40408120

[bib4] Beznosyk, A., Quax, P., Coninx, K., & Lamotte, W. (2011). Influence of network delay and jitter on cooperation in multiplayer games. *Proceedings of the 10th International Conference on Virtual Reality Continuum and Its Applications in Industry*. Hong Kong, China, December 11–12, 2011 (pp. 351–354), 10.1145/2087756.2087812.

[bib5] Botzer, L., & Karniel, A. (2013). Feedback and feedforward adaptation to visuomotor delay during reaching and slicing movements. *European Journal of Neuroscience**,* 38(1), 2108–2123, 10.1111/ejn.12211. 23701418

[bib6] Cámara, C., de la Malla, C., López-Moliner, J., & Brenner, E. (2018). Eye movements in interception with delayed visual feedback. *Experimental Brain Research**,* 236, 1837–1847, 10.1007/s00221-018-5257-8. 29675715 PMC6010481

[bib7] Caserman, P., Martinussen, M., & Göbel, S. (2019). Effects of end-to-end latency on user experience and performance in immersive virtual reality applications. In: *Joint International Conference on Entertainment Computing and Serious Games* (pp. 57–69). Cham: Springer International, 10.1007/978-3-030-34644-7_5.

[bib8] Cattan, E., Perrier, P., Bérard, F., Gerber, S., & Rochet-Capellan, A. (2018). Adaptation to visual feedback delays on touchscreens with hand vision. *Experimental Brain Research**,* 236, 3191–3201, 10.1007/s00221-018-5368-2. 30191261

[bib9] Claypool, M., & Claypool, K. (2006). Latency and player actions in online games. *Communications of the ACM**,* 49(11), 40–45, 10.1145/1167838.1167860.

[bib10] Cunningham, D. W., Billock, V. A., & Tsou, B. H. (2001). Sensorimotor adaptation to violations of temporal contiguity. *Psychological Science**,* 12(6), 532–535, 10.1111/1467-9280.d01-17150. 11760144

[bib11] Cunningham, D. W., Chatziastros, A., Von der Heyde, M., & Bülthoff, H. H. (2001). Driving in the future: temporal visuomotor adaptation and generalization. *Journal of Vision**,* 1(2), 3, 10.1167/1.2.3. 12678604

[bib12] Davis, J., Smyth, C., & McDowell, K. (2010). The effects of time lag on driving performance and a possible mitigation. *IEEE Transactions on Robotics**,* 26(3), 590–593, 10.1109/TRO.2010.2046695.

[bib13] de la Malla, C., López-Moliner, J., & Brenner, E. (2012). Seeing the last part of a hitting movement i3s enough to adapt to a temporal delay. *Journal of Vision**,* 12(10), 4, 10.1167/12.10.4. 22961221

[bib14] de la Malla, C., López-Moliner, J., & Brenner, E. (2014). Dealing with delays does not transfer across sensorimotor tasks. *Journal of Vision**,* 14(12), 8, 10.1167/14.12.8. 25301016

[bib15] Ebbinghaus, H. (1885). *Über das gedächtnis: Untersuchungen zur experimentellen psychologie*. Berlin: Duncker & Humbolt.

[bib16] Foulkes, A. J. M., & Miall, R. C. (2000). Adaptation to visual feedback delays in a human manual tracking task. *Experimental Brain Research**,* 131, 101–110, 10.1007/s002219900286. 10759175

[bib17] Georg, J. M., Feiler, J., Hoffmann, S., & Diermeyer, F. (2020). Sensor and actuator latency during teleoperation of automated vehicles. *2020 IEEE Intelligent Vehicles Symposium (IV)*. Las Vegas, Nevada, October 19–November 13, 2020 (pp. 760–766). IEEE, 10.1109/IV47402.2020.9304802.

[bib18] Goonatilake, R., & Bachnak, R. A. (2012). Modeling latency in a network distribution. *Network and Communication Technologies**,* 1(2), 1, 10.5539/nct.v1n2p1.

[bib19] Hadjiosif, A. M., Abraham, G., Ranjan, T., & Smith, M. A. (2024). Subtle visual latency can profoundly impair implicit sensorimotor learning. bioRxiv, 10.1101/2024.03.14.585093. PMC12062458 40341213

[bib20] Helmholtz H (1867) Handbuch der physiologischen Optik. In: G. Karsten (Ed.), Allgemeine Encyklopädie der Physik (vol 9). Leipzig: Leopold Voss.

[bib21] Hing, J. T., Sevcik, K. W., & Oh, P. Y. (2009). Improving unmanned aerial vehicle pilot training and operation for flying in cluttered environments. *2009 IEEE/RSJ International Conference on Intelligent Robots and Systems*. St. Louis, Missouri, October 11–15, 2009 (pp. 5641–5646). IEEE, 10.1109/IROS.2009.5354080.

[bib22] Hosseini, E. A., Nguyen, K. P., & Joiner, W. M. (2017). The decay of motor adaptation to novel movement dynamics reveals an asymmetry in the stability of motion state-dependent learning. *PLoS Computational Biology**,* 13(5), e1005492, 10.1371/journal.pcbi.1005492. 28481891 PMC5440062

[bib23] Itkonen, T. H., Pekkanen, J., Lappi, O., Kosonen, I., Luttinen, T., & Summala, H. (2017). Trade-off between jerk and time headway as an indicator of driving style*.* *PLoS One**,* 12(10), e0185856, 10.1371/journal.pone.0185856. 29040291 PMC5645088

[bib24] JASP Team. (2023). JASP (Version 0.17.2). Available at: https://jasp-stats.org/home/front-page-0-17-2-1png/.

[bib25] Jota, R., Ng, A., Dietz, P., & Wigdor, D. (2013). How fast is fast enough? a study of the effects of latency in direct-touch pointing tasks. In *Proceedings of the Sigchi Conference on Human Factors in Computing Systems*. Paris, France, April 27–May 2, 2013 (pp. 2291–2300), 10.1145/2470654.2481317.

[bib26] Knelange, E. B., & López-Moliner, J. (2019). Decreased temporal sensorimotor adaptation due to perturbation-induced measurement noise*.* *Frontiers in Human Neuroscience**,* 13, 46, 10.3389/fnhum.2019.00046. 30837854 PMC6382734

[bib27] Körding, K. P., & Wolpert, D. M. (2004). Bayesian integration in sensorimotor learning. *Nature**,* 427(6971), 244–247, 10.1038/nature02169. 14724638

[bib28] Korenberg, A. T., & Ghahramani, Z. (2002). A Bayesian view of motor adaptation. *Current Psychology of Cognition**,* 21(4/5), 537–564.

[bib29] Krakauer, J. W., Hadjiosif, A. M., Xu, J., Wong, A. L., & Haith, A. M. (2019). Motor learning. *Comprehensive Physiology**,* 9(2), 613–663, 10.1002/j.2040-4603.2019.tb00069.x. 30873583

[bib30] Kolekar, S., De Winter, J., & Abbink, D. (2020). Human-like driving behaviour emerges from a risk-based driver model. *Nature Communications**,* 11(1), 4850, 10.1038/s41467-020-18353-4. PMC7525534 32994407

[bib31] Lambert, E., Romano, R., & Watling, D. (2019). Optimal path planning with clothoid curves for passenger comfort. *Proceedings of the 5th International Conference on Vehicle Technology and Intelligent Transport Systems (VEHITS 2019)*. Heraklion, Greece, May 3–5, 2019 (Vol. 1, pp. 609–615). SciTePress, 10.5220/0007801806090615.

[bib32] Lappi, O. (2014). Future path and tangent point models in the visual control of locomotion in curve driving. *Journal of Vision**,* 14(12), 21, 10.1167/14.12.21. 25761280

[bib33] Leow, L. A., De Rugy, A., Marinovic, W., Riek, S., & Carroll, T. J. (2016). Savings for visuomotor adaptation require prior history of error, not prior repetition of successful actions*.* *Journal of Neurophysiology**,* 116(4), 1603–1614, 10.1152/jn.01055.2015. 27486109 PMC5144718

[bib34] Liu, R., Kwak, D., Devarakonda, S., Bekris, K., & Iftode, L. (2017). Investigating remote driving over the LTE network. In *Proceedings of the 9th International Conference on Automotive User Interfaces and Interactive Vehicular Applications*. Oldenburg, Germany, September 24–27, 2017 (pp. 264–269), 10.1145/3122986.3123008.

[bib35] Mehta, B., & Schaal, S. (2002). Forward models in visuomotor control. *Journal of Neurophysiology**,* 88(2), 942–953, 10.1152/jn.2002.88.2.942. 12163543

[bib36] Miall, R. C., & Jackson, J. K. (2006). Adaptation to visual feedback delays in manual tracking: Evidence against the Smith Predictor model of human visually guided action. *Experimental Brain Research**,* 172, 77–84, 10.1007/s00221-005-0306-5. 16424978

[bib37] Morehead, J. R., Qasim, S. E., Crossley, M. J., & Ivry, R. (2015). Savings upon re-aiming in visuomotor adaptation. *Journal of Neuroscience**,* 35(42), 14386–14396, 10.1523/JNEUROSCI.1046-15.2015. 26490874 PMC4683692

[bib38] Morehead, J. R., Taylor, J. A., Parvin, D. E., & Ivry, R. B. (2017). Characteristics of implicit sensorimotor adaptation revealed by task-irrelevant clamped feedback. *Journal of Cognitive Neuroscience**,* 29(6), 1061–1074, 10.1162/jocn_a_01108. 28195523 PMC5505262

[bib39] Murdock, B. B. Jr, & Cook, C. D. (1960). On fitting the exponential. *Psychological Reports**,* 6(1), 63–69, 10.2466/pr0.1960.6.1.6.

[bib40] Nagengast, A. J., Braun, D. A., & Wolpert, D. M. (2010). Risk-sensitive optimal feedback control accounts for sensorimotor behavior under uncertainty. *PLoS Computational Biology**,* 6(7), e1000857, 10.1371/journal.pcbi.1000857. 20657657 PMC2904762

[bib42] Nikravesh, A., Choffnes, D. R., Katz-Bassett, E., Mao, Z. M., & Welsh, M. (2014). Mobile network performance from user devices: A longitudinal, multidimensional analysis. In: *International Conference on Passive and Active Network Measurement* (pp. 12–22). Cham: Springer, 10.1007/9783-319-04918-2_2.

[bib43] Noguera Cundar, A., Fotouhi, R., Ochitwa, Z., & Obaid, H. (2023). Quantifying the effects of network latency for a teleoperated robot. *Sensors**,* 23(20), 8438, 10.3390/s23208438. 37896531 PMC10611222

[bib44] Rahimian, P., Plumert, J. M., & Kearney, J. K. (2021). The effect of visuomotor latency on steering behavior in virtual reality. *Frontiers in Virtual Reality*, 2, 727858, 10.3389/frvir.2021.727858.

[bib46] Ribeiro, R., Ramos, J., Safadinho, D., Reis, A., Rabadão, C., Barroso, J., … Pereira, A. (2021). Web AR solution for UAV pilot training and usability testing. *Sensors**,* 21(4), 1456, 10.3390/s21041456. 33669733 PMC7922183

[bib47] Rohde, M., & Ernst, M. O. (2016). Time, agency, and sensory feedback delays during action*.* *Current Opinion in Behavioral Sciences**,* 8, 193–199, 10.1016/j.cobeha.2016.02.029.

[bib48] Rohde, M., Van Dam, L. C., & Ernst, M. O. (2014). Predictability is necessary for closed-loop visual feedback delay adaptation. *Journal of Vision**,* 14(3), 4, 10.1167/14.3.4. 24599942

[bib49] Sheridan, T. B. (1993). Space teleoperation through time delay: Review and prognosis*.* *IEEE Transactions on Robotics and Automation**,* 9(5), 592–606, 10.1109/70.258052.

[bib50] Smith, M. A., Ghazizadeh, A., & Shadmehr, R. (2006). Interacting adaptive processes with different timescales underlie short-term motor learning. *PLoS Biology**,* 4(6), e179, 10.1371/journal.pbio.0040179. 16700627 PMC1463025

[bib51] Shadmehr, R., Smith, M. A., & Krakauer, J. W. (2010). Error correction, sensory prediction, and adaptation in motor control. *Annual Review of Neuroscience**,* 33(1), 89–108, 10.1146/annurev-neuro-060909-153135. 20367317

[bib52] Sober, S. J., & Sabes, P. N. (2003). Multisensory integration during motor planning. *Journal of Neuroscience**,* 23(18), 6982–6992, 10.1523/JNEUROSCI.23-18-06982.2003. 12904459 PMC6740676

[bib53] Sober, S. J., & Sabes, P. N. (2005). Flexible strategies for sensory integration during motor planning. *Nature Neuroscience**,* 8(4), 490–497, 10.1038/nn1427. 15793578 PMC2538489

[bib54] Stornig, A., Fakhreddine, A., Hellwagner, H., Popovski, P., & Bettstetter, C. (2021). Video quality and latency for UAV teleoperation over LTE: A study with ns3. *2021 IEEE 93rd Vehicular Technology Conference (VTC2021-Spring)*. Virtual conference, April 15–28, 2021 (pp. 1–7). IEEE, 10.1109/VTC2021Spring51267.2021.9448676.

[bib55] Thomas, J. A., & Walton, D. (2007). Measuring perceived risk: Self-reported and actual hand positions of SUV and car drivers. *Transportation Research Part F: Traffic Psychology and Behaviour**,* 10(3), 201–207, 10.1016/j.trf.2006.10.001.

[bib56] Thoroughman, K. A., & Shadmehr, R. (2000). Learning of action through adaptive combination of motor primitives. *Nature**,* 407(6805), 742–747, 10.1038/35037588. 11048720 PMC2556237

[bib57] Van Rooij, A. J., Schoenmakers, T. M., Vermulst, A. A., Van Den Eijnden, R. J., & Van De Mheen, D. (2011). Online video game addiction: Identification of addicted adolescent gamers. *Addiction**,* 106(1), 205–212, 10.1111/j.1360-0443.2010.03104.x. 20840209

[bib58] Vozar, S., Storms, J., & Tilbury, D. M. (2018). Development and analysis of an operator steering model for teleoperated mobile robots under constant and variable latencies. *Robotica**,* 36(2), 167–186, 10.1017/S0263574716000679.

[bib59] Waltemate, T., Senna, I., Hülsmann, F., Rohde, M., Kopp, S., Ernst, M., … Botsch, M. (2016). The impact of latency on perceptual judgments and motor performance in closed-loop interaction in virtual reality. *Proceedings of the 22nd ACM Conference on Virtual Reality Software and Technology*. Munich, Germany, November 2–4, 2016 (pp. 27–35), 10.1145/2993369.2993381.

[bib60] Warburton, M., Mon-Williams, M., Mushtaq, F., & Morehead, J. R. (2023). Measuring motion-to-photon latency for sensorimotor experiments with virtual reality systems. *Behavior Research Methods**,* 55(7), 3658–3678, 10.3758/s13428-022-01983-5. 36217006 PMC10616216

[bib61] Wei, K., & Kording, K. (2009). Relevance of error: what drives motor adaptation? *Journal of Neurophysiology**,* 101(2), 655–664, 10.1152/jn.90545.2008. 19019979 PMC2657056

[bib62] Welch, R. B. (2013). *Perceptual modification: Adapting to altered sensory environments*. New York: Elsevier.

[bib63] Widrow, B., McCool, J. M., Larimore, M. G., & Johnson, C. R. (1976). Stationary and nonstationary learning characteristics of the LMS adaptive filter. *Proceedings of the IEEE**,* 64(8), 1151–1162, 10.1109/PROC.1976.10286.

[bib64] Xu, S., Perez, M., Yang, K., Perrenot, C., Felblinger, J., & Hubert, J. (2014). Determination of the latency effects on surgical performance and the acceptable latency levels in telesurgery using the dV-Trainer simulator. *Surgical Endoscopy**,* 28(9), 2569–2576, 10.1007/s00464-014-3504-z. 24671353

[bib65] Zarahn, E., Weston, G. D., Liang, J., Mazzoni, P., & Krakauer, J. W. (2008). Explaining savings for visuomotor adaptation: linear time-invariant state-space models are not sufficient. *Journal of Neurophysiology**,* 100(5), 2537–2548, 10.1152/jn.90529.2008. 18596178 PMC2585408

